# A DNA Origami Bubble Blower for Liposome Production

**DOI:** 10.1021/acsomega.4c05297

**Published:** 2024-10-17

**Authors:** Gerrit D. Wilkens, Piotr Stępień, Yusuke Sakai, Md. Sirajul Islam, Jonathan G. Heddle

**Affiliations:** † Malopolska Centre of Biotechnology, Jagiellonian University, Gronostajowa 7A, 30-387 Krakow, Poland; ‡ Postgraduate School of Molecular Medicine, Żwirki i Wigury 61, 02-091 Warsaw, Poland

## Abstract

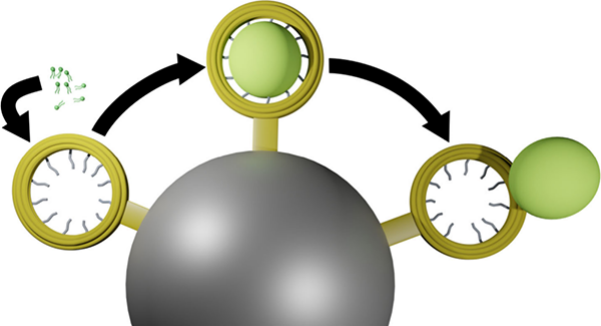

While liposomes are commonly produced on the industrial scale with diameters in the hundreds of nanometers to micrometer size range, the variation from the mean diameter can be significant. In nature, functional liposome-like systems can be as small as tens of nanometers in diameter but reproducible synthetic production at this size scale is not easily achievable. Here we outline the development of a DNA origami “bubble blower”—a nanoscale ring able to seed and constrain liposome formation. The bubble blower has the potential to be employed in a reusable fashion for production of nanometric liposomes. It improves currently available DNA origami liposome seeding techniques by expanding the range of compatible detergents and introducing solid support integration with potential for semiautomated laboratory scale production.

## 1 Introduction

Cells contain multiple lipid-bound structures in the form of small vesicles. These are involved in numerous biological processes such as transport, exocytosis, and compartmentalization.^1^ Synthetically produced vesicles and liposomes are valuable tools for basic research, for example for characterization of membrane proteins, are a core component for the bottom-up construction of artificial cells,^2^ and have gained great utility as drug delivery systems,^3^ their use in delivery of mRNA in COVID-19 vaccines being a notable recent example.^4^

Commonly employed methods for generation of nanosized vesicles are mainly based on mechanical dispersion of rehydrated lipid films by extrusion^5^ and sonication.^6^ Microfluidics has also been widely used to create small and homogeneous liposomes, albeit usually requiring in-house bespoke devices.^7^ An intriguing and novel alternative is to guide lipid self-assembly on DNA origami “exoskeleton” nanotemplates to create size and shape defined vesicular lipid structures.^8,9^ There, the size and the shape of the DNA template dictates the size and shape of the seeded liposomes, which, by virtue of the homogeneity of DNA origami, allows for consistent preparation of sub 100 nm liposomes with lower size variation compared to standard mechanical approaches. Interestingly, DNA origami can remain attached on the assembled liposome structure, meaning that it can act as a modular platform or pegboard that can be used to organize liposomes at specific locations and with specific separation distances, which has been used to study nonvesicular lipid transport.^10^

During template guided liposome assembly, lipid molecules conjugated to the DNA origami structure act as seeding domains that promote growth of liposomes from detergent solubilized lipids upon detergent removal by dialysis. Consequently, a purification step is required to remove excess free lipid molecules from the final product. Typically, this has been achieved using ultracentrifugation in density gradients where separation is due to the buoyancy of the sample. However, an ultracentrifugation step is a potential limitation for the adaptation of the technique for high-throughput use. Moreover, depending on the DNA origami structure used, considerable optimization of the ultracentrifugation gradient is required to ensure sufficient separation between free lipids (i.e., liposomes formed in the solution and not bound to DNA origami) and the DNA origami-liposome structures.

**Figure 1:**
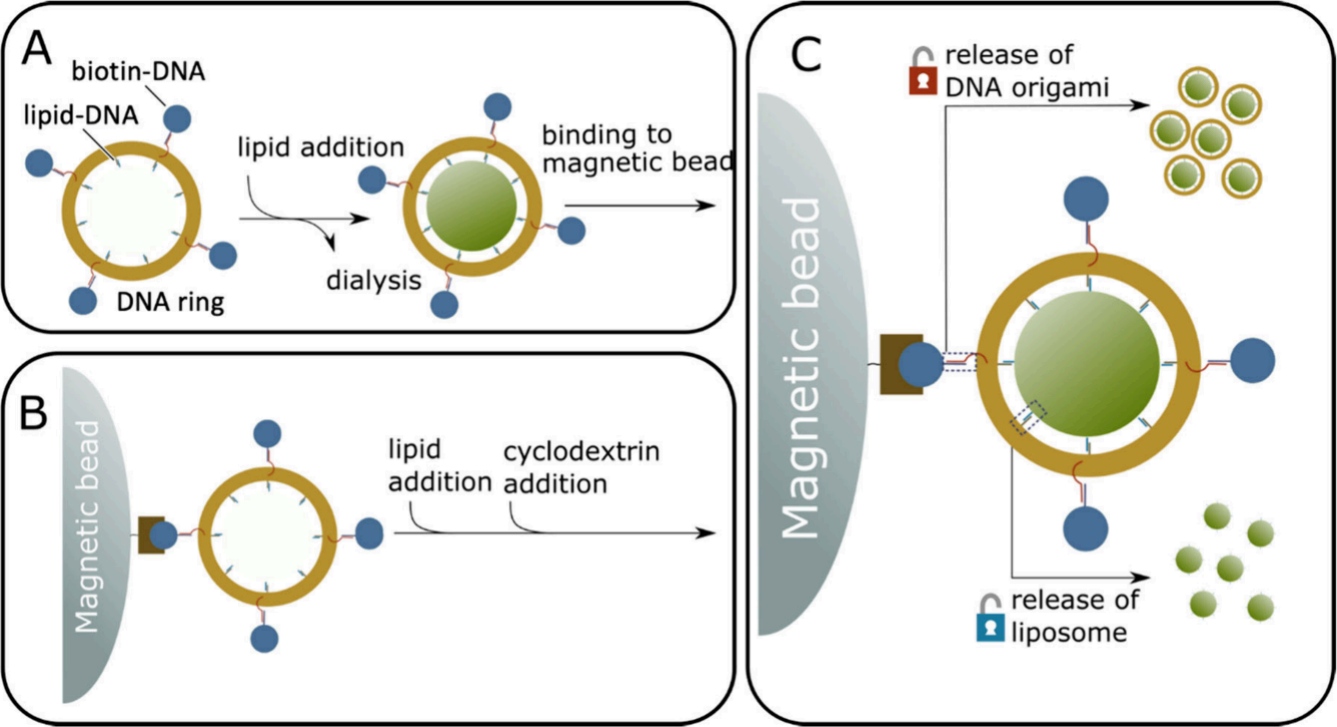
Proposed scheme of liposome generation and purification of a DNA origami template on a solid support. (A) The DNA origami has staple extensions that can be hybridized with complementary lipid or biotin modified DNA strands. Note that in both cases 12 strand extensions are available at evenly spaced positions on the DNA origami ring. For clarity not all strand extension are shown in the schematic. To produce liposomes, detergent solubilized lipids are supplied to a DNA origami template that is either in solution or (B) bound to magnetic beads using a biotin–streptavidin interaction between a biotinylated DNA modification of the DNA origami and a streptavidin coated magnetic bead. Then, to initiate lipid vesicle formation the detergent is removed either via dialysis or by complexation reaction with cyclodextrin. (C) After liposome formation is completed, the DNA origami can be captured with a magnetic bead if not already bound and the excess lipids can be washed away by exchanging the buffers using pipetting. The formed lipid vesicles or the origami-liposome *complex* can be released by a strand displacement reaction that displaces either (i) the biotin strand from the origami or (ii) the lipid-modified strand.

In this work, we tested the feasibility of a magnetic bead-based method for purification of DNA origami templated liposomes (Figure 1), inspired by previous works on purification of protein–DNA origami conjugates by attachment to magnetic beads.^11^ We further hypothesized that similarly to the way that solid-phase synthesis of peptides and nucleic acids represented a breakthrough technology,^12^ the DNA origami mediated solid-phase (magnetic bead) based approach we demonstrate here for liposome production/purification assembly may also prove useful upon further refinement and development. In contrast to the previously published methods, we show that liposome formation on DNA origami templates can also be modulated by exploiting cyclodextrin-detergent host–guest chemistry (Figure 1B) that has previously proven useful for detergent removal from solutions^13^ and for reconstituting membrane proteins into liposomes.^14^ In contrast to dialysis, this enables the use of hard-to-dialyze detergents with low critical micelle concentrations (CMC) such as the ubiquitous *n*-dodecyl-β-d-maltoside (DDM).

## 2 Results

### 2.1 DNA Origami Design and Bead Attachment

We adopted and modified a DNA origami ring design with a 46 nm diameter inner cavity from Yang et al.^8^ and used it as a template for size-defined liposome growth. Modifications included (i) addition of biotin receiver strand extensions on the DNA ring perpendicular to the plane of the ring that allowed biotin strand binding and (ii) adoption of the scaffold sequence to our in-house produced scaffold based on the plasmid pScaf-3024.t (see scaffold production in the Supporting Information).

The complete DNA origami ring design had a total of 12 receiver extensions facing toward the cavity of the DNA ring that could be hybridized with complementary lipid-modified handle strands as well as 12 receiver strands for hybridization with biotinylated-handle strands. Both lipid receiver strands and biotinylated-handle strands were designed to have a 6 nt toehold sequence to enable strand displacement of their respective handles by addition of strands that had a fully complementary sequence to the receiver strands (Figure S1). We use the term “invader strands” to denote these strands.

The modified DNA ring was assembled in a 36 h thermal annealing ramp and purified using rate-zonal centrifugation.^8^ Assembly was verified using agarose gel electrophoresis and transmission electron microscopy (Figure S2). Next, we modified the DNA origami rings with biotin handles and successfully demonstrated an efficient attachment of the DNA origami to magnetic beads and its release triggered by strand displacement using agarose gel electrophoresis (Figure S3).

### 2.2 Templated Liposome Production in Solution

To generate liposomes enclosed inside the DNA ring cavity (origami-liposome complexes), we produced a lipid-modified DNA using thiol-maleimide click coupling (Figure S4) and hybridized the strands to complementary sequences facing toward the cavity of the DNA rings.

**Figure 2:**
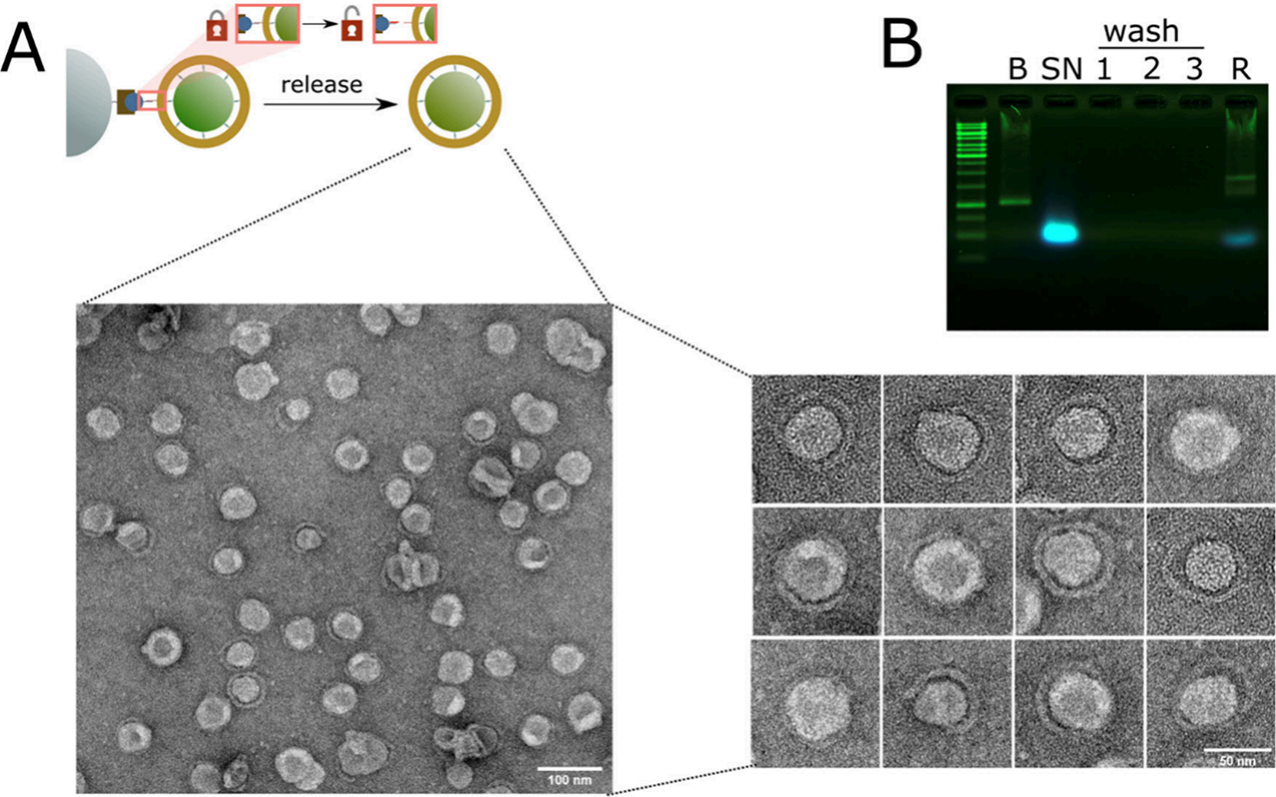
DNA ring formation and magnetic bead purification in solution. (A) Negative stain TEM image of assembled DNA-ring liposome structures assembled in solution and purified by binding to and release from magnetic beads. Scale bars: 100 and 50 nm for enlarged and cropped images, respectively. (B) Agarose gel electrophoresis of DNA origami ring liposome complex during the purification process. Fluorescence of rhodamine-labeled lipids is shown in blue and EtBr-stained DNA is shown in green. Abbreviations: B, DNA ring before liposome formation; SN, supernatant after magnetic-bead attachment; 1–3, wash fractions; R, released DNA-ring samples. Note: the agarose gel and running buffer were supplemented with 0.05% SDS to solubilise the liposomes and allow the samples to enter the gel.

Next, origami-liposome complexes were produced as reported^8^ where DNA rings having lipid-DNA strands were mixed with octyl β-d-glucopyranoside (OG) solubilized lipids, after which the detergent was removed during an overnight dialysis step. Importantly, in contrast to previous work, we did not employ ultracentrifugation for purification of the origami-liposome complexes but attached the DNA ring to a magnetic bead using streptavidin–biotin binding. After the attachment, we removed excess lipid by repeated washing of the beads. Then, by addition of appropriate invader strands that remove the biotinylated strands from the DNA origami, we released the DNA origami-liposome complex from the beads (Figure 2). Unlike ultracentrifugation-based approaches, our method does not exclude empty or partially formed structures. Bearing this in mind, we quantitated such contaminants by analyzing a total of 210 particles from TEM images with visual inspection, showing that 73% of all observed particles were correctly formed liposomes bound inside the DNA origami rings.

**Figure 3:**
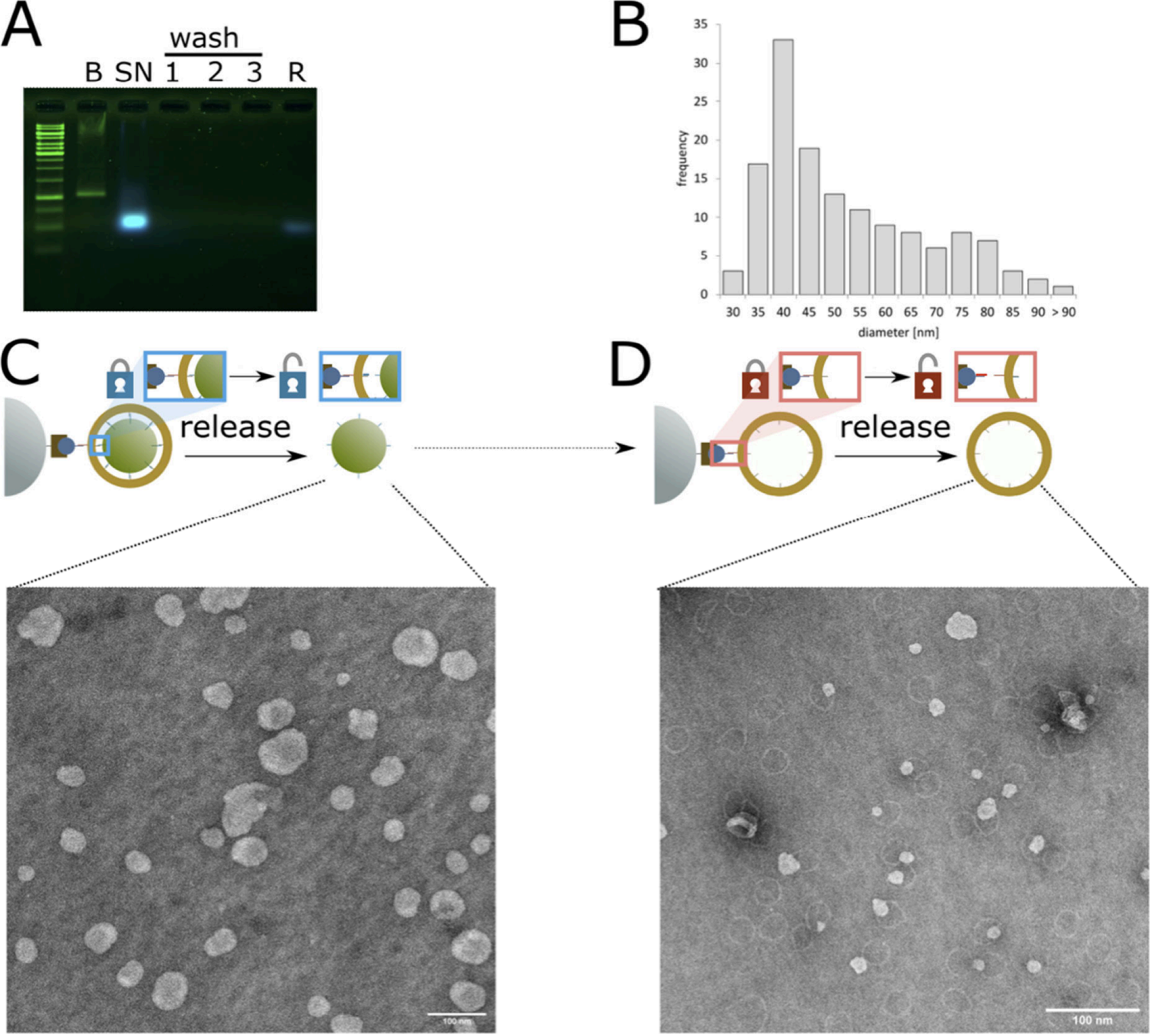
Liposome release from magnetic beads. (A) Agarose gel electrophoresis of liposomes during the purification process. Fluorescence of rhodamine-labeled lipids is shown in blue, and EtBr-stained DNA is shown in green. Abbreviations: B, DNA ring before liposome formation; SN, supernatant after bead attachment; 1–3, wash fractions; R, released DNA-ring samples. Note: the agarose gel and running buffer were supplemented with 0.05% SDS to solubilise the liposomes and allow the samples to enter the gel. (B) Histogram of the diameter of released liposomes measured from TEM images, *N* = 140. (C) Negative stain TEM of liposomes released from magnetic beads. (D) Negative stain TEM of DNA origami rings released from magnetic beads, after liposomes were released from the DNA origami rings Scale bars: 100 nm. We estimated the diameter of the liposome products from TEM images (Figure 3B, Figures S5 and S6), which showed that the majority of the liposomes were in the intended range of 30–50 nm while a few larger liposomes approximately double the size were also present.

After successful demonstration of the magnetic bead purification process, we triggered the release of liposomes from the bead-attached DNA ring templates and compared it with the triggered release of the full DNA ring liposome complexes or of empty DNA ring complexes. To elute the liposomes from the DNA origami template, another strand displacement reaction was used, enabling decoupling of the lipid-DNA conjugate from the DNA origami ring. Agarose gel electrophoresis and negative stain TEM of liposomes confirmed their release from magnetic beads (Figures 3A,C). The remaining DNA origami ring template was separately collected by another targeted release reaction. In some cases, these showed that smaller particles (presumably parts of the liposome) remained attached to the DNA ring, likely due to the lower accessibility of the connecting strands for strand displacement (Figure 3D). The fact that, post liposome release, intact DNA rings can be released and recovered, strongly suggests that, if not released, they are present on the magnetic beads, meaning that they would be available for a second round of liposome generation.

**Figure 4:**
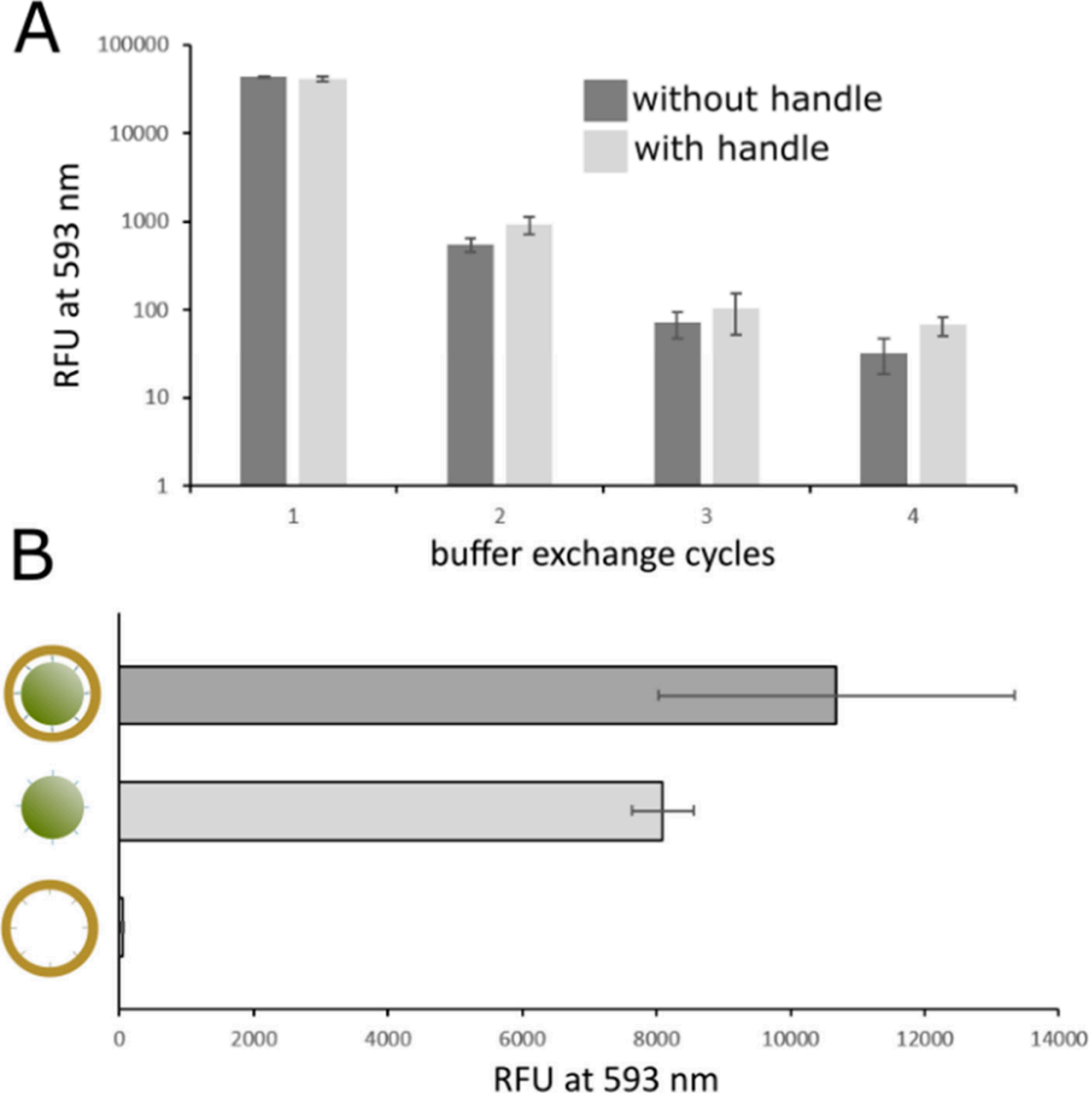
Removal of excess lipids from magnetic beads and release of liposomes and origami-liposome complex. (A) Relative fluorescence of rhodamine-labeled lipids in wash fractions from magnetic beads. Separate liposome formation reactions on DNA origami ring templates were carried out in solution using DNA origami rings without and with lipid handles which should be incapable or capable of templating liposome formation, respectively (dark gray and light gray, respectively). Following liposome formation, DNA origami structures were captured using magnetic beads, followed by four cycles of buffer exchange to remove excess lipids that did not form DNA origami-attached liposomes. Fluorescence intensity of free lipids of wash steps 1–4 is displayed: extinction, 560 nm; emission, 593 nm; *n* = 3. (B) Results of targeted release of DNA-ring-liposome complexes, the lipid component alone from within DNA-ring-liposome complexes, or DNA rings without lipid handles. The *X*-axis shows relative fluorescence of rhodamine-labeled lipids from the released products: extinction, 560 nm; emission, 593 nm; *n* = 3.

To see how effectively free lipids are removed from the magnetic beads and to compare the release of full origami-liposome complexes and liposomes alone we monitored the rhodamine fluorescence of washes and released fractions (Figure 4). The quantity of rhodamine-labeled lipid in solution was monitored over four cycles of buffer exchange (Figure 4A) as well as in the liposome elution step triggered by invader strands (Figure 4B). To estimate nonspecific binding to the beads, we compared the DNA origami rings with and without inclusion of the lipid-modified DNA which help to form and bind liposomes (Figure 4A).

The free lipid was efficiently transferred to wash fractions in both cases. Additionally, releasing liposomes alone and liposome within DNA origami rings showed comparable efficiency (Figure 4B), suggesting that liposomes constrained within the DNA rings remained stable on the magnetic bead surface during the washing steps. The slightly lower efficiency of liposome release compared to release of DNA-ring constrained liposomes can be attributed to the previously noted residual lipids remaining on the DNA ring (Figure 3D). No lipid-release was apparent from a control reaction in which liposome production was carried out without the use of lipid-DNA modified handle strands.

### 2.3 Integration on Solid Support

Next, we attempted liposome formation directly on the solid support. To this end, we first attached biotinylated and lipidated DNA rings on the magnetic beads. After the attachment we exchanged the buffer from the beads to remove potentially unbound DNA origami and subsequently added lipids solubilized using DDM. DDM was chosen as a representative low-CMC, high-aggregation-number detergent which cannot be easily removed using dialysis. Moreover, from a practical standpoint dialysis of a magnetic beads resin slurry tends to be problematic due to handling issues and the sedimentation of the resin To mitigate both problems and to remove the DDM, we used β-cyclodextrin, which forms inclusion complexes with the detergent. This procedure abrogates the detergency of DDM and breaks up its micelles (Figure S7), which allows for its subsequent removal along with free lipids via washing. Additionally, it is worth noting that while detergent dialysis is usually slow, cyclodextrin mediated detergent removal is rapid and can be precisely tuned by sequential addition of cyclodextrin to the detergent solution.

**Figure 5:**
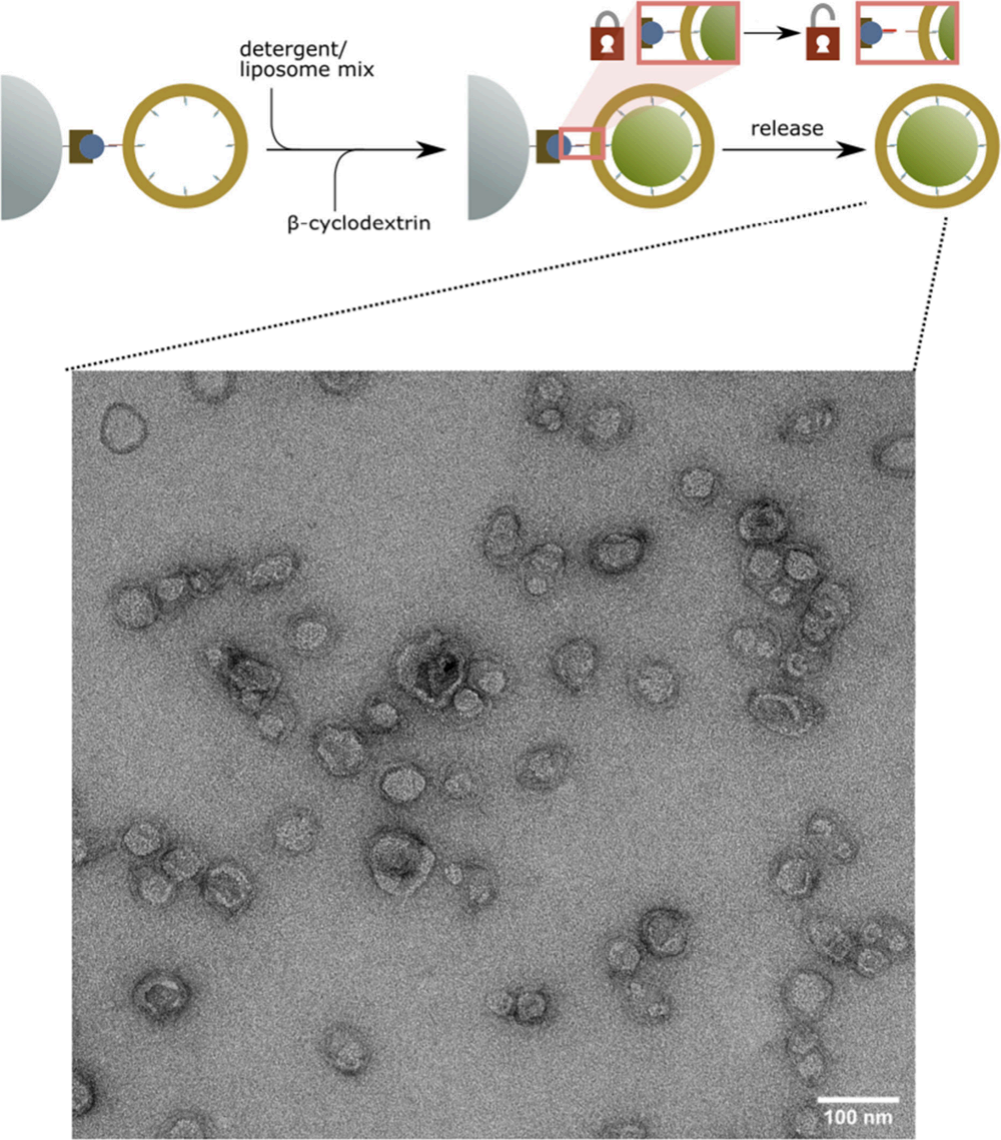
Negative stain TEM of DNA origami liposome complexes prepared on solid support. Scale bar: 100 nm.

After the DDM removal by cyclodextrin, origami-liposome complexes were eluted from the beads by addition of the appropriate invader strand as was the case for the in-solution assembly of DNA ring liposomes. The results achieved were comparable to the in-solution assembly procedure (Figure 5).

## 3 Discussion

Lipid bilayers are of vital importance in living cells, as they provide a physical barrier delineating the cell and the external environment and, within cells, separate functional compartments.^1^

DNA nanostructures have been used in a number of ways to actuate, functionalize, or control the shape and size of liposomes.^15^ Interaction of liposomes with DNA nanostructures can be achieved by either attaching the DNA nanostructure to preformed liposomes^16^ or, alternatively, growing liposomes on DNA nanostructure templates.^8−10,17^

During DNA nanostructure guided self-assembly, lipid molecules attached to the rigid structure act as aggregation points where detergent micellized lipids can bind during detergent removal. To date, DNA origami guided liposomes have only been formed in solution, using a dialysis step to remove the detergent used to solubilize lipid molecules, followed by ultracentrifugation to remove all byproducts formed during the detergent removal step.^8−10,17^ This setup is disadvantageous in the sense that (i) ultracentrifugation may limit the scalability and throughput of liposome production, (ii) the requirement for dialysis restricts the use of detergent to those having a low CMC, as large micellar detergents are difficult to remove,^14^ while also limiting easy integration into possible continuous-flow systems, and (iii) depending on the detergent used, dialysis can be slow which might pose a significant challenge when seeking to reconstitute easily aggregating membrane proteins into the liposomes.

In this work we aimed to increase the applicability of DNA nanostructure templated liposome formation by developing a solid-phase-based method for liposome production on DNA origami templates. In our approach, DNA origami ring structures are tethered to a solid support using biotin–streptavidin interaction. We successfully showed that liposomes are formed directly on the solid-surface-attached DNA origami and can be released using a strand displacement reaction. The liposomes produced in this way retain externally displayed seeding DNA strands which may be useful for future derivatization. Alternatively, they can be removed by simple enzymatic digestion as has been previously demonstrated.^8^ Lastly, detergent removal based on complexation with β-cyclodextrin vastly expands the selection of detergents compatible with this method and by extension compatibility with a wider range of membrane proteins in future applications. We hope that this approach may prove useful for scalable production of small, modifiable liposomes.

## 4 Conclusions

In summary, we have here shown the feasibility of DNA magnetic beads to act as anchor points that allow easy purification of DNA bound lipid vesicles from surrounding medium and larger free-floating liposomes. Moreover, we show that lipid vesicles can be grown directly while being anchored on magnetic beads and eluted from them using a simple DNA strand displacement reaction. Second, we demonstrate that cyclodextrin-mediated detergent complexing is compatible with the DNA origami template-based assembly technique which, as cyclodextrin can be easily titrated, opens the way for fine-tuned, controllable liposome assembly and even membrane protein insertion in the future.

## 5 Materials and Methods

### 5.1 DNA Origami Assembly

DNA origami samples were assembled by mixing 50 nM scaffold and 200 nM staple strands in buffer containing 5 mM Tris, 1 mM EDTA, and 10 mM MgCl_2_. Samples were then annealed in a thermocycler using the following temperature ramp: heating to 80 °C for 10 min, 79 °C, −1 °C/1 min for 19 cycles, 60 °C, −1 °C/100 min for 20 cycles and 40 °C, −1 °C/10 min for 17 cycles and purified using rate-zonal centrifugation using a 15–45% (w/v) glycerol gradient.

### 5.2 Templated Liposome Production in Solution

DNA origami rings at a 10 nM concentration were modified with lipid modified and biotinylated handle strands by addition of 3× and 1.5× molar excess over the respective receiver strands in liposome formation buffer (25 mM HEPES, pH 7.4, 150 mM KCl, 10 mM MgCl_2_) supplemented with 1% (w/v) OG. This was followed by 30 min incubation at 24 °C. Next, 5 μL of 15 mM lipid mix containing 15% DOPS, 0.8% Rho-PE, and 84.2% DOPC were added to the DNA origami solution. The origami solution was incubated for 1 h at room temperature with slight shaking and then filled to 120 μL by addition of 60 μL 1× liposome formation buffer supplemented with 0.67% OG, transferred to a dialysis cassette (Pur-A-Lyzer Mini 12000, Sigma-Aldrich), and dialyzed for 16 h at room temperature against 1.5 L liposome formation buffer. After dialysis, the solution was recovered from the dialysis device and added to 20 μL of a magnetic bead solution that was prewashed with liposome formation buffer. The DNA origami ring liposome solution was incubated for 1 h at 24 °C to attach the DNA origami structures to the magnetic beads. After attachment, the supernatant containing mostly free lipids was removed and the beads were washed three times with 100 μL of liposome formation buffer. After the washing steps, magnetic beads were resuspended in 20 μL liposome formation buffer, to which 2.8 μL of a 5 μM stock of the appropriate release strand was added (6× molar excess over the receiver strands on the DNA origami), that either displaced the lipid-modified DNA from the DNA origami ring, releasing liposomes or displaced the biotinylated DNA, releasing DNA origami ring liposome complexes from the magnetic beads.

### 5.3 Templated Liposome Production on Magnetic Beads

For the on-bead origami-liposome complex formation, DNA origami rings were hybridized with modifying handles first as described in the in-solution formation methods and then added to 20 μL of a magnetic bead solution that was prewashed with liposome formation buffer containing 10 mM DDM. The DNA origami ring liposome solution was incubated for 1 h at 24 °C to attach the DNA origami structures to the magnetic beads. Then 5 μL of 15 mM lipid mix containing 15% DOPS, 0.8% Rho-PE, and 84.2% DOPC were added to the DNA origami solution followed by an incubation for 30 min at room temperature. The detergent was then sequestered by addition of β-cyclodextrin in two steps. First 40 μL of 10 mM β-cyclodextrin was added and incubated for 10 min followed by another addition of 80 μL of β-cyclodextrin and 10 min incubation. Excess lipids were washed away, and origami-liposome complex release was triggered as in the case of the in-solution liposome production.

### 5.4 Fluorescence Measurements

Fluorescence of Rho-PE containing liposomes was measured at room temperature using a RF-6000 spectrofluorophotometer (Shimadzu) with excitation at 560 nm and emission from 570 to 800 nm at a scan speed of 2000 nm/min. Excitation and emission bandwidth were set to 5 nm and sensitivity to low.

### 5.5 Transmission Electron Microscopy

For negative stain transmission electron microscopy, grids were glow discharged for 60 s at 8 mA on a Leica EM ACE200 instrument. 4 μL of the sample was deposited on the grid incubated for approximately 1 min and blotted away using filter paper. The grid was stained for 1 min using 2% uranyl acetate solution. Images were acquired using a JEOL TEM 2100HT instrument (Jeol Ltd.) used at 80 kV accelerating voltage. Images were taken by a 4k × 4k camera (TVIPS) microscope.

## Supporting Information Available

Information on materials used, notes on design of receiver, handle, and invader DNA strands including Nupack analysis and list of functional DNA strands used, protocol for ssDNA scaffold production, protocol for DNA origami assembly and purification with agarose gel electrophoresis and negative-stain transmission electron microscopy data, protocol for lipid-DNA conjugate production, agarose gel electrophoresis analysis of binding of DNA origami ring to magnetic beads, polyacrylamide gel electrophoresis of preparation of lipid-DNA conjugate, negative-stain transmission electron microscopy analysis of DNA origami formed liposomes, thin layer chromatography analysis of host–guest chemistry of β-cyclodextrin and N-dodecyl-β-maltoside, and details of the DNA origami ring design including staple and scaffold DNA sequences (PDF)
