# Supplementary material for: A DNA Origami Bubble Blower for Liposome Production
Source: ACS Omega. 2024 Oct 17;9(43):43609–15. doi: 10.1021/acsomega.4c05297 (PMC11525534; doi:10.1021/acsomega.4c05297)
Supplement: Supplementary file 1 — ao4c05297_si_001.pdf [file ao4c05297_si_001.pdf]

Supplementary information

A DNA origami bubble blower for liposome production

Gerrit D. Wilkens[1,2,†], Piotr Stępień[1 ‡], Yusuke Sakai[1, §], Md. Sirajul Islam[1, ¶], and Jonathan G. Heddle,[1, ‡,]*

[1]Malopolska Centre of Biotechnology, Jagiellonian University, Gronostajowa 7A, 30-387 Krakow, Poland
[2]Postgraduate School of Molecular Medicine, Żwirki i Wigury 61, 02-091, Warsaw, Poland

*E-mail: Jonathan.g.heddle@durham.ac.uk

[†]Current address: Université de Montpellier, CNRS, INSERM, Centre de Biologie Structurale, F-34000 Montpellier, France

[‡]Current Address : Department of Biosciences, Durham University, Durham, DH1 3LE, UK.

[§]Current address: RIKEN Center for Biosystems Dynamics Research, 6-2-3, Furuedai, Suita, Osaka, 565-0874, Japan

[¶]Current address: School of Biotechnology and Biomolecular Sciences, University of New South Wales, Kensington, NSW 2052, Australia.

Index

Materials 3

Design of receiver, handle and invader strands 3

Scaffold production 5

DNA origami production and purification 5

Lipid-DNA conjugate production 6

TEM image analysis of DNA origami formed liposomes 9

Cadnano strand diagram of DNA origami ring 12

List of staple strands 13

Scaffold sequence 14

Supplementary references 15

Materials

1,2-dioleoyl-sn-glycero-3-phosphocholine (DOPC), 1,2-dioleoyl-sn-glycero-3-phospho-L-serine (DOPS), and 1,2-dioleoyl-sn-glycero-3-phosphoethanolamine-N-(lissamine rhodamine B sulfonyl) (Rhod PE) were purchased from Avanti Polar lipids and stored at -20 °C in chloroform.

1,2-dioleoyl-sn-glycero-3-phosphoethanolamine-N-(p-maleimidophenyl)butyramide] (18:1 MBP PE) was purchased from Avanti lipids and stored dissolved in chloroform at 10 mg/ml in glass vials at -80 °C. Lipid mixes were prepared by mixing different lipid species at appropriate conditions. Lipids were dried under argon stream followed by vacuum for 2 h. Lipid films were resuspended in liposome formation buffer (25 mM HEPES, 150 mM KCl, 10 mM $MgCl_{2,}$ pH 7.4) and directly used.

DNA staple strands were purchase from Integrated DNA Technologies in 96 well plates normalised to a concentration of 200 µM in TE buffer. DNA strands were premixed and stored at – 20 °C until further usage.

5'-[Thio/C6] CGTGAGGTGTGGTAGAT-3' was synthesised and HPLC purified by Sigma Aldrich.

Design of receiver, handle and invader strands

Receiver strands are defined as extensions on the DNA origami that hybridise with complementary lipid modified DNA. Handle strands are defined as (lipid or biotin) modified strands that hybridise to receiver strands. Invader strands are defined as strand displacing DNA that binds to a toehold on the receiver strands and displace the handle strands. Strands were designed by hand and initially examined for undesired cross-binding to other strands in the system by Nupack[1] as shown in Supp. Fig. 1. Strand sequence for the lipid receiver/ handle system is adapted from Yang et al[2]. For strand sequences see Supp. Table 1.

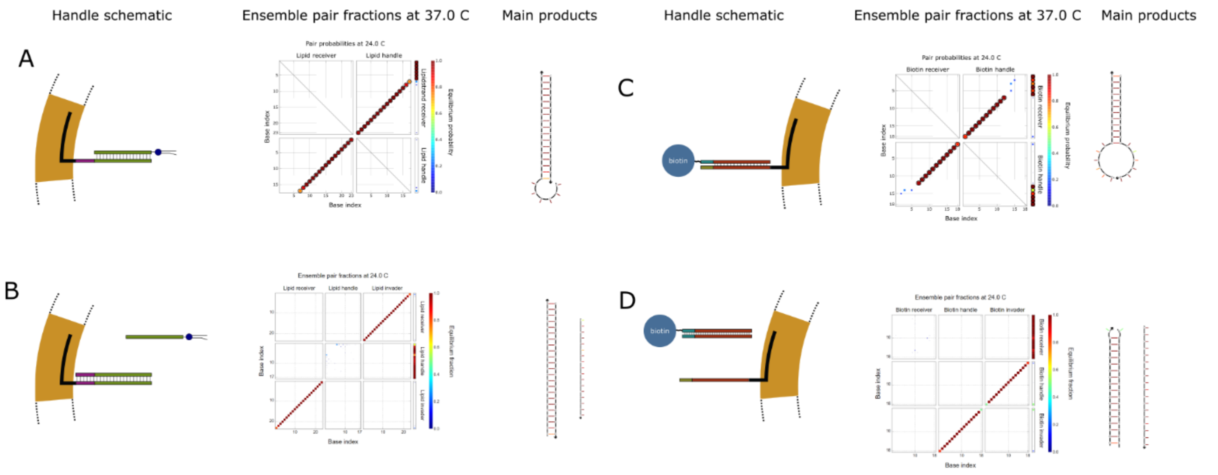

Figure S1. Binding of strands was estimated by Nupack at equimolar strand concentrations (1 µM) at a temperature of 24°C and a maximal complex size of 2 for A) lipid receiver/ handle B) lipid receiver/handle/ invader C) biotin receiver/ handle D) biotin anti receiver/handle/invader. Schematics at the right-hand side of each panel depict the intended binding situation given the present strands.

Table S1. Strand sequences for: receiver extensions on the DNA origami; modified handle strands and invader strands Toehold domains are depicted in bold and underlined.

Name	Sequence (5' to 3' end)	Function
Lipid receiver	[staple sequence]-**CACATC**ATCTACCACACCTCACG	Extension on staple strands of DNA origami that can bind the lipid modified handle strand
Lipid handle	[DOPE/C6]-CGTGAGGTGTGGTAGAT	Lipidated handle strand for seeding liposome formation inside DNA origami ring
Lipid invader	CGTGAGGTGTGGTAGATGATGTG	Invader strand binding to lipid anti handle strand via a toehold on the lipid anti handle strand thereby removing the lipid handle from the DNA origami structure
Biotin receiver	[Staple Sequence]-**CACTCT**CCTATCACTACC	Extension on staple strands of DNA origami that can bind the biotinylated handle strand
Biotin handle	[Btn]GGTAGTGATAGGCGTTCT	Biotinylated handle strand that binds to DNA origami extensions and attaches the DNA origami to streptavidin coated surfaces
Biotin handle invader	AGAACGCCTATCACTACC	Invader strand binding to biotin handle strand via a toehold on the biotin handle strand thereby removing the attachment of DNA origami structure to magnetic beads by freeing up the biotin receiver strands

Scaffold production

Helper plasmid HP17_KO7 was a gift of Hendrik Dietz (addgene #120346). The phagemids pScaf and pScaf-3024.1 were gifted by Shawn Douglas (addgene #111401 and 111404 respectively). We subcloned the insert of pScaf-3024.1 to pScaf using BamHI and KpnI cutting sites which we termed pScaf-3024.t. For Scaffold production we transformed *E. coli* XL1-blue with HP17_KO7 and pScaf-3024.1t. Scaffold production was essentially carried out as described previously[3], with the exception that 6 ml lysis buffer were used per 100 ml culture with culture volume ranging from 100 ml to 1 L.

DNA origami assembly and purification

The DNA origami ring was adopted from Yang et al.[2] with small adaptions of the staple routing to allow extensions for attachment of the biotinylated DNA strand extensions and change of strand sequences to adjust the scaffold sequence to the pScaf-3024 scaffold sequence. Changes were made using CaDNANo software[4]. Staple strands were obtained from Integrated DNA Technologies. DNA origami rings were assembled in a one-pot reaction by mixing of 50 nM scaffold with 200 nM staples in assembly buffer containing 5 mM Tris, 1 mM EDTA (pH 8) and 10 mM $MgCl_2$ and subjected to the following temperature ramp in a thermocycler: heating to 80 °C for 10 min, 79 °C, -1 °C/1 min for 19 cycles, 60 °C, -1 °C/ 100 min for 20 cycles and 40 °C, -1 °C / 10 min for 17 cycles. A semi-linear glycerol gradient was prepared in 13 x 51 mm tubes (Beckman Coulter) by layering 400 µL glycerol solutions from 15-45 % in liposome formation buffer (25 mM HEPES, 150 mM KCl, 10 mM $MgCl_2$) in 5 % increments into the tube, starting with the lightest solution first and loading each following solution carefully to the bottom of the tube. 400 µL of assembled DNA origami solution was concentrated to 100 µL using a 0.5 ml 10 kDa cut-off filter, carefully laid on top of the gradient and centrifuged at 50,000 rpm and 4 °C for 180 min in a Optima MAX XP Ultracentrifuge (Beckman Coulter) using a MSL-50 swinging bucket rotor. After the centrifugation, the solution was fractionated into 14 to 15 fractions starting from the top. Fractions were analysed on an agarose gel. Pure DNA origami fractions were buffer exchanged against liposome formation buffer and concentrated using 0.5 ml Amicon filters with a cut-off of 30 kDa. DNA origami concentration was assessed by UV-Vis spectrophotometry using a Nanodrop ND-1000.

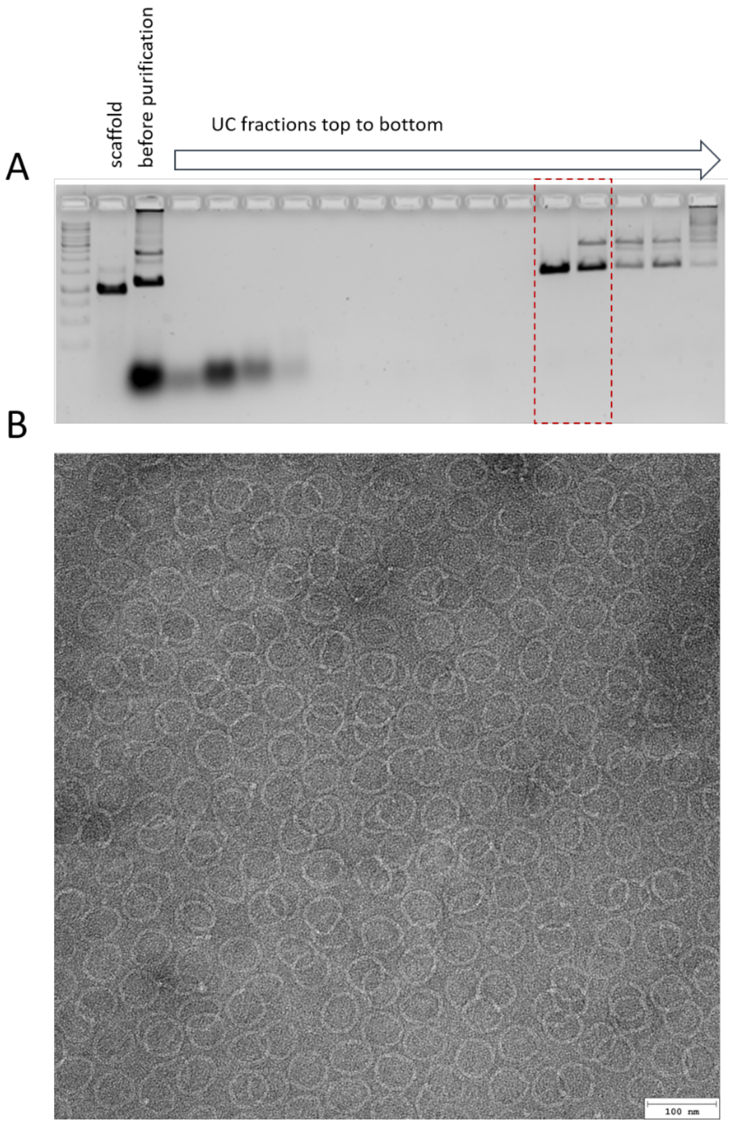

Figure S2. Purification of assembled DNA origami rings by ultracentrifugation. A) Agarose gel electrophoresis of ultracentrifugation fractions. Pure fractions that were pooled and used for further experiments are marked by a red square. B) TEM image of pooled DNA origami ring structures after agarose gel analysis.

Lipid-DNA conjugate production:

Thiol-C6 modifed DNA oligonucleotide: 5'-[Thio/C6] CGTGAGGTGTGGTAGAT-3' was synthesised and HPLC purified by Sigma Aldrich. 1,2-dioleoyl-sn-glycero-3-phosphoethanolamine-N-(p-maleimidophenyl)butyramide] (18:1 MBP PE) was purchased from Avanti lipids and stored dissolved in chloroform at 10 mg/ml in glass vials at -80 °C. Lipid modification was carried out following a previously reported protocol with minor modifications [2]. 40 µL of 10 mg/ ml 18:1 MPB PE stored in chloroform (0.4 µmol, 40 x excess of lipid) was dried in a glass vial under argon stream followed by vacuum for 2 h. 400 µM DNA oligo was reduced for 30 min at room temperature in a buffer containing 25 mM HEPES, 140 mM KCl and 25 mM Tris(2-carboxyethyl)phosphine, pH 7.2 in a volume of 25 µL. The reaction was transferred to a 3 kDa cut-off amicon filtered and mixed with 100 µL conjugation

buffer without TCEP followed by subsequent concentration to the original volume, which was repeated twice. Next, the freshly reduced DNA was added to the glass vial together with 5 µL of 20 % octyl-beta glucopyranoside (OG). The lipid film was carefully dissolved by pipetting, and argon was briefly blown into the vial. The reaction was incubated for 2 h at 37°C. Coupling efficiency was assessed by running 5 µL of the DNA diluted to 1 µM on a 10 % PAGE gel in 1 x TBE supplemented with 0.1 % SDS in gel and running buffer for 1 h at 100 V. The reaction efficiency was 95 % as estimated from gel profiles and considered high enough to be used without further purification.

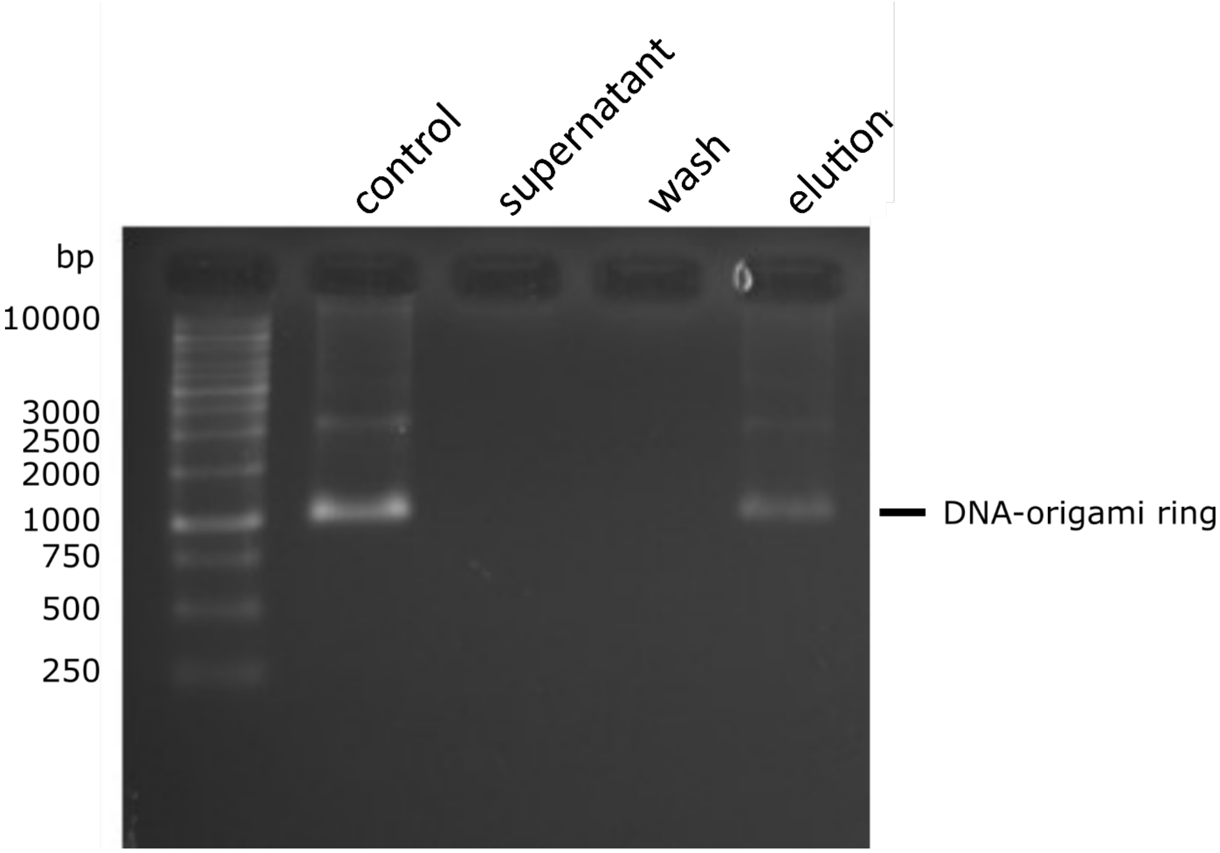

Figure S3. Magnetic bead binding and release of the DNA origami ring. Control: DNA origami ring before adding to the magnetic beads. Supernatant: Supernatant of the magnetic bead solution after addition and binding of the DNA origami rings, wash: wash fractions of the magnetic beads, elution: supernatant of the magnetic beads after addition of the biotin-invader strand.

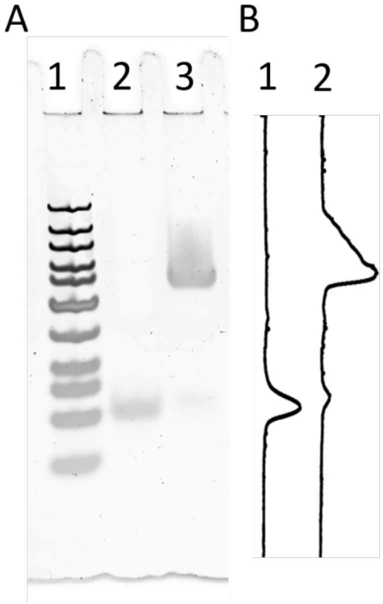

Figure S4. Production of lipid modified DNA. A) Gel analysis of lipid conjugate. 1) Size marker, 2) unmodified SH-DNA, 3) lipid-DNA conjugate. 5 µL of each sample diluted to 1 µM was run in a 10 % PAGE gel in 1 x TBE buffer supplemented with 0.1 % SDS and stained with Sybr Gold. Ladder: GeneRuler Ultra Low Range DNA Ladder (Thermofisher Scientific) B) Gel-lane profile of 1) SH-DNA and 2) modified DNA.

TEM image analysis of DNA origami formed liposomes

A

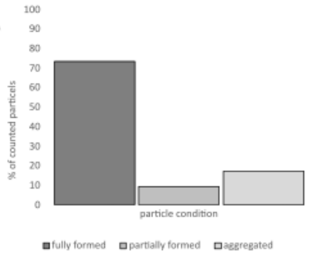

TEM	fully formed	partially formed	aggregated	
B	44	4	10	
C	34	5	11	
D	36	6	9	
E	40	5	6	
Total	154	20	36	210

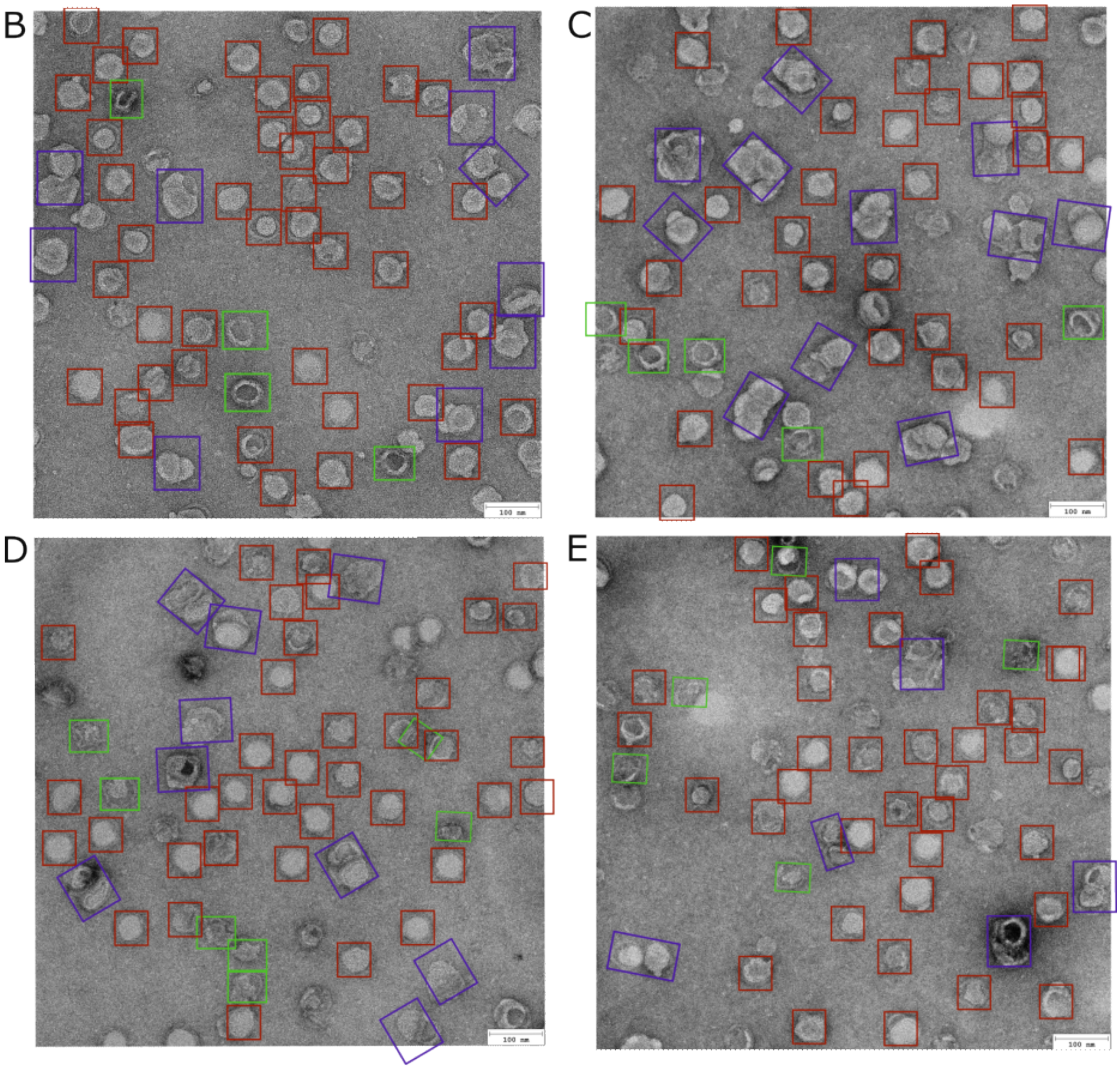

Figure S5. Frequency estimation of DNA origami liposome complex condition. Red square: well-formed particles, green square: empty DNA origami rings/ incompletely formed liposomes. Purple square; aggregated structures. Fully formed liposomes were defined as discrete single structures roughly circular, consistent with the appearance of an approximately spherical particle in TEM with a continuous outer circumference. Where the outer circumference appeared discontinuous, these discrete particles were classified as “partially formed”. Where more than one circular structure was partially or fully overlayed on one or more other such structures they were classified as “aggregated” Note that aggregated structures may have been fully formed and aggregated only on the TEM grid meaning that the % fully formed may be an underestimate

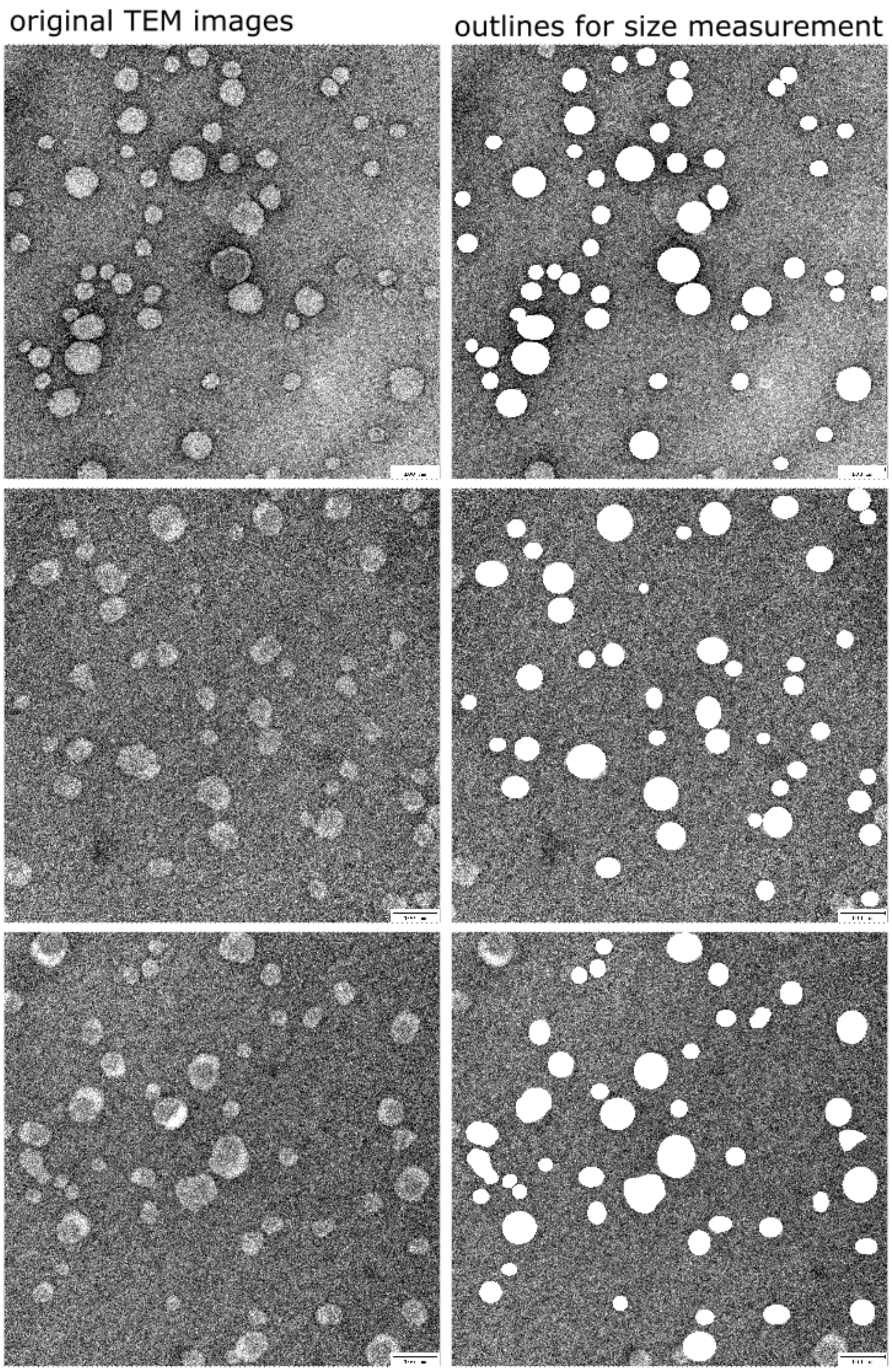

Figure S6. Size measurement from TEM images. The outline of liposomes on TEM images were approximated with circles or in some cases with freehand drawing tool in imageJ to obtain an estimate the area of the liposomes which was used to approximate the diameter of the liposomes by assuming a spherical particle.

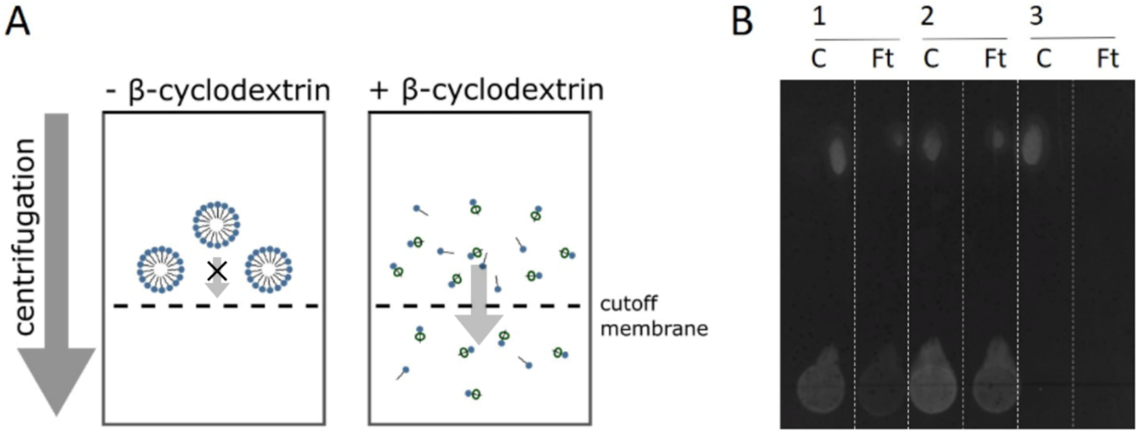

Figure S7. Host-guest chemistry of β-cyclodextrin and N-Dodecyl-β-maltoside (DDM). A) Schematic representation of the effect of β-cyclodextrin addition to DDM. Above the critical micelle concentration (CMC) DDM forms micelles that are too big to pass though cut-off membranes. β-cyclodextrin forms complexes with the detergent and breaks up DDM micelles. Individual detergent molecules are then able to pass through the cut-off filter. B) Thin-layer chromatography of DDM-cyclodextrin complexes. Spotted TLC solutions were obtained by concentrating 1) DDM- β-cyclodextrin in a final 1:1 ratio 2) DDM- β-cyclodextrin in a final 1:3 ratio or 3) DDM without β-cyclodextrin for details of incubation see material and methods. C: concentrate in the filter FT: flow through.

Cadnano strand diagram of DNA origami ring structure

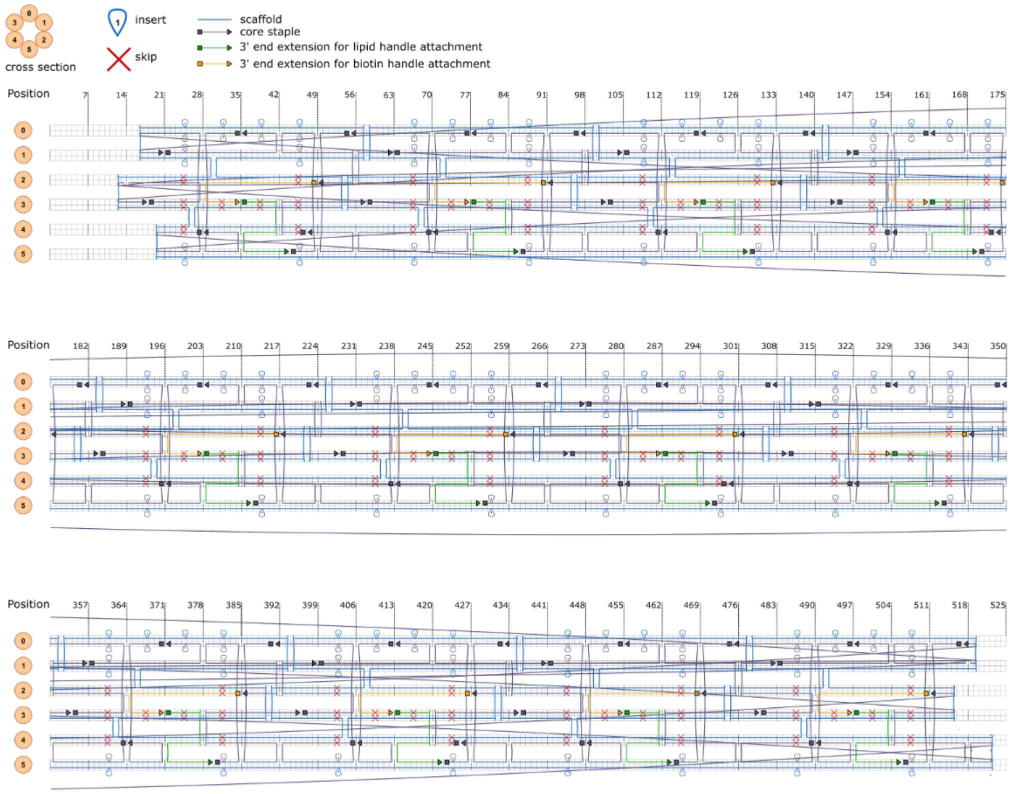

List of staple strands

"Start" and "end" denote the beginning and end of the staples in the strand diagram in the format of number of helix[position on helix]

Start	End	Sequence	Function
1[21]	0[35]	GGGGAAAGCCGGCGAACGCCAAAGGG	3' extension for lipid handle extenson sequence is: CACATCATCTACCACACCTCACG
1[63]	0[77]	TAGCCCGAGATAGGGTTGCCCGGCAA	
1[10]	0[11]	TTGTCCACCTCAGATTGGCGCTTCAG	
1[14]	0[16]	CACTTCACCCTGGGCGCGGCCCAGTT	
1[18]	0[20]	AGATGCTGACCTTGTCTTTCTCGTCC	
1[23]	0[24]	ATGCCTGCGTGGGTAGTCAGGTCCAG	
1[27]	0[28]	GAAACAGACAGAGTTGATGTTTGCAG	
1[31]	0[32]	ATGGGAACAGAGAAGGGTGCATCTGT	
1[35]	0[37]	TGGATAATGTCGAGAATCTTGTTCAG	
1[39]	0[41]	GGTGAGGTAACACTTGTCGTCCAGGC	
1[44]	0[45]	TGAACTCGTCGAAGCAACTGGCGCTC	
1[48]	0[49]	CGGAACAGCTGCTTCAGGTTATTGTC	
3[35]	5[43]	AGCCCCGCACTAAGCTTCACCA	3' end extension handle to attach biotinylated DNA. Extension sequence is TTCACTCTCCTATCACTACC
3[77]	5[85]	AATCCCTCGTAGGCTCCAGAGA	
3[11]	5[12]	GATAGGGTGTTCTCACTCCCAT	
3[16]	5[16]	CCCTGACACCCACTCTTGATCT	
3[20]	5[21]	AGTTCGTTGAGGTACCTGCTTC	
3[24]	5[25]	GATGGTCTGGCATGAGTTCAAT	
3[28]	5[29]	TAATGTCTCCCTCTTGTGCAGT	
3[32]	5[33]	TCGGCCACATCTTAGAGCCTGG	
3[37]	5[37]	CTGCTTGGTGTGCACCGCTCTT	
3[41]	5[42]	CCATCTGACCGTAGGACGGTCT	
3[45]	5[46]	AGCACATGGATCTGGTGTCAGG	
3[49]	5[50]	TGGATGGACTTCGTCTGGAGAG	
4[46]	3[57]	TAAACGATTTAGAGCACCC	core staples
4[17]	3[18]	ACAGGTGCTTCGCGGCTCT	
4[29]	3[30]	ACTTGCTTAGACTTCTTGG	
4[42]	3[43]	GCTGTCATAGCCTGTAGTG	
3[18]	1[20]	GGCGCGTCTGCGCTTAATGCGCTATAACC	
3[58]	1[62]	AAATCAAGTATACTACGTGAACCAAATATCA	
3[102	1[104	GGCCATTTAAAAGGTGATCCAACCAAGTA	
3[144	1[146	GGAACAGATCGCGAGGCAGACCTCTTTCT	
3[184	1[188	GGGAGTCCTATAGCTTCATGGTGCCTCCTTC	
3[228	1[230	TTCGAGGTGGGCTCCGCGACCATCGTCTG	
3[270	1[272	GCTTCTCCAAGAAGCGAGGGAAATATCGT	
3[310	1[314	ACAAGGGCATGCAGGTTTCGACGTCTGGGCC	
3[354	1[356	GTGCAGGTGCACCAGCTGTGTCTTTACTG	
3[396	1[398	TCAACTGTCGGTCGGACTTCATCCCTGAA	
3[436	1[440	CCTTAACGCCGCCCAGCAGACCTCGGACACG	
3[48]	1[48]	CCTGTGGTCACCCAACAGCTCGCATTCAC	
0[96]	3[10]	ATTAAATTACCTGCTGCTGGAACTCTTTATAAATCAATTCATT	
0[13]	3[14]	TTTCCTACACAGCGATCGGGTGACAGGATAACAATCTGCAAGC	
0[22]	3[22]	GACTGCGCGCTCATCTCTGAATATGGGCGAGTTTCCTGATTGG	
0[26]	3[26]	TGTCATGAACACACGTTGAGGCTGCGCAGCAGTGAGTCGCTGC	
0[348]	3[353]	ACGCACATGTGGCAATTGCGTTCTCCAAGCACAGGATAATCTG	
0[390]	3[395]	CTCTGTCTGCCTGCCCAATCTTCGGTGACCACGTCGAAGAACC	
0[474]	3[479]	ATTCTTCTTGACAGTCCTGGATGGTCGATTCCTCCAGGACGGT	
0[516]	3[17]	TTTACCAGCATCTCGGAATAGAGGATGGACATAGCCACTACAG	
0[54]	4[47]	GCGGGCCAGTCTTGAGGTGCCG	
0[180]	4[173]	CCACGAGGATAATAGTGTGAAC	
0[306]	4[299]	TCACGGATGTGAAAATGGCACA	
0[432]	4[425]	TTCCTTCTCTCAGCCAGTGCCA	
4[27]	0[517]	CCGGATCCTCCCTTTGAAGGGATTCATGGTCACGCTGCGCGTCC	
4[69]	0[55]	AATTTTTGGGGTCGCACAGTGCTGCTCTGCGAAAAACCGTCGGA	
4[11]	0[97]	CGTCTTGGAATCCCGCCTGATGGGCCCAAAACTCCTCAAACACT	

4[15]	0[139]	AAATCCAGCCAGTTGTTCATACAGTTCCCACCGAACAGCAGCAC	
4[195]	0[181]	ATCATAGTCGATCCTCATAGTTAGCCTCTGGTTGACGATGTTGG	
4[237]	0[223]	ATTAAACCCACTTGCTCGTCATACTCCACCTGTTGAATCTGAAG	
4[279]	0[265]	TTCTCCAGGAACTTCTTTAATCAGAGCGTCTCCAGCAGGTCATC	
4[321]	0[307]	CTCCTTATTCTTGCTTTGACATGGTATTTTCTTCATCTCCAGAA	
4[363]	0[349]	TTGAGTGATCTTTTCTCATTTGCATTCTGTCGATGTTAGTAGTC	
4[405]	0[391]	CCCTTCTTCCAGGAGGAGATTTCCTGAACGCTGTCAACCATGGA	
4[447]	0[433]	GATCTCTGTCTTAACCCCAGTCGTAATGTCATCACAGTTGACAT	
4[489]	0[475]	GCTTCTTCAATGCGATTGAAGATGGCCATCCGGAGTTGGAGAAG	
5[44]	2[49]	CCTTTCGCTAGGGCAAGGGAAGAAAGCGATCTTGAC	
5[86]	2[91]	GTGCTCGAACGTGGGTTTGGAACAAGAGTTCAAGAA	
5[128]	2[133]	CCTCTGGAGGCGGTATCACAGAATGCTCCCATATAT	
5[170]	2[175]	GATTAGTGGTATCGTTTCAGGTCAGCAGCGGATGGT	
5[212]	2[217]	AAAGCGAGGAGGCAAGTATGGTGAGTCCTAATTCCC	
5[254]	2[259]	ACCAGGGATGCTGCCGATTGTCCTTCTTGGGTGCGA	
5[296]	2[301]	CTGTGAATGGATTGCTTCTTGAGGTGAGATATCATC	
5[338]	2[343]	ACGACATCCTTCAGGAGAGCTGCGTCCGGTCCTGTG	
5[380]	2[385]	GTTCAGGTTACCTGTTCAGCTCCAGAATAGTTGTTA	
5[422]	2[427]	TGAGGGGGTACTCAGTAAACGAGCTTGCTCGGTCAA	
5[464]	2[469]	AGTTCTCCCATGGAACTTAACGAGGCCAACACCTGT	
5[506]	2[511]	AACTTTTGCGGGATTTCTGTCTTCCTCCACGCTGGG	
0[34]	4[28]	GTGTAGCGGTGTATTATCGGAA	
0[76]	4[70]	GTCAAAGGCAGCATTTACCCCG	
0[118]	4[112]	TTCATCAGCAATCACCCGCGGA	
0[160]	4[154]	CAGCAGGTAATGCTTGCCCCAC	
0[202]	4[196]	CGCCCACCCTCAAACTCTGCAA	
0[244]	4[238]	TCATCCACCTTCTTTCTCTGCA	
0[286]	4[280]	GCCCAAGATCAAATCCAAGGAC	
0[328]	4[322]	CCAGGGTCCAGCCTCCGGTAAA	
0[370]	4[364]	GAAGATGTGTGTTGTACTTGGA	
0[412]	4[406]	CTGGAACTCCTCTCATGGGTTG	
0[454]	4[448]	CGATTCCTCTGCTGAGCATAGA	
0[496]	4[490]	ATGAAGATCGAGCTTGCGATCA	
1[21]	0[35]	ACCACACCCGTGGCGAGAAAGGGCTGGCAA	
1[63]	0[77]	GGGCGATGGCAGTGTTGTTCCAACTCCAAC	
1[105]	0[119]	GGTCTTAGTGAGCTTGACAGTCCGTGCCAA	
1[147]	0[161]	CCAGAGATGTTGAGTTCAGATCGCAACTCT	
1[189]	0[203]	CAATCCTTGACAAAGCCTTTATTCTATTGT	
1[231]	0[245]	CTCTTTGGGCAAGGTTGTGACGGGAAATCT	
1[273]	0[287]	CTCCGATGAAACCAGCAAACAGGTTGACTG	
1[315]	0[329]	AGATCGTTCACCACAGCTCCCTCAGCTGCT	
1[357]	0[371]	CTCCAATTTGAGTGCTTTCAGTGGTCCTCG	
1[399]	0[413]	CGCAACAGGTAGTGAGTGGAGTTAGGTGTA	
1[441]	0[455]	AGGACCTGCCCCCAAGCTCCACCTTAACGT	
1[483]	0[497]	CTCCACTTCCTTGTCTGGGAGTTCATTGTG	

Scaffold sequence

Scaffold is from Nafisi et al. [3] For the readers convenience the scaffold sequence is reproduced below:

GGATCCACGCGCCCTGTAGCGGCGCATTAAGCGCGGCGGGTGTGGTGGTTACGCGCAGCGTGACCGCTACACTTGCCAGCGCCCTAGCG
CCCGCTCCTTTCGCTTTCTTCCCTTCCTTTCTCGCCACGTTCGCCGGCTTTCCCCGTCAAGCTCTAAATCGGGGGCTCCCTTTAGGGTT
CCGATTTAGTGCTTTACGGCACCTCGACCCCAAAAAACTTGATTTGGGTGATGGTTCACGTAGTGGGCCATCGCCCTGATAGACGGTTT
TTCGCCCTTTGACGTTGGAGTCCACGTTCTTTAATAGTGGACTCTTGTTCCAAACTGGAACAACACTCAACCCTATCTCGGGCTATTCT
TTTGATTTATAAGGGATTTTGCCGATTTCGGGGTACCTACGAAGAGTTCCAGCAGGGATTCCAAGAAATGGCCAATGAAGATTGGATCA
CCTTTCGCACTAAGACCTACTTGTTTGAGGAGTTTCTGATGAATTGGCACGACCGCCTCAGGAAAGTGGAGGAGCATTCTGTGATGACT

```
GTCAAGCTCCAATCTGAGGTGGACAAATATAAGATTGTTATCCCTATCCTGAAGTACGTCCGCGGAGAACACCTGTCACCCGATCACTG
GCTGGATCTGTTCCGCTTGCTGGGTCTGCCTCGCGGCACATCTCTGGAGAAACTGCTGTTCGGTGACCTGCTGAGAGTTGCCGATACCA
TCGTGGCCAAGGCTGCTGACCTGAAAGATCTGAACTCACGCGCCCAGGGTGAAGTGACCATCCGCGAAGCACTCAGGGAACTGGATTTG
TGGGGCGTGGGTGCTGTGTTCACACTGATCGACTATGAGGACTCCCAGAGCCGCACCATGAAGCTGATCAAGGATTGGAAGGACATCGT
CAACCAGGTGGGCGACAATAGATGCCTCCTGCAGTCCTTGAAGGACTCACCATACTATAAAGGCTTTGAAGACAAGGTCAGCATCTGGG
AAAGGAAACTCGCCGAACTGGACGAATATTTGCAGAACCTCAACCATATTCAGAGAAAGTGGGTTTACCTCGAACCAATCTTTGGTCGC
GGAGCCCTGCCCAAAGAGCAGACCAGATTCAACAGGGTGGATGAAGATTTCCGCAGCATCATGACAGATATCAAGAAGGACAATCGCGT
CACAACCTTGACTACCCACGCAGGCATTCGCAACTCACTGCTGACCATCCTGGACCAATTGCAGAGATGCCAGCGCAGCCTCAACGAGT
TCCTGGAGGAGAAGCGCAGCGCCTTCCCTCGCTTCTACTTCATCGGAGACGATGACCTGCTGGAGATCTTGGGCCAGTCAACCAATCCA
TCCGTGATTCAGTCTCACCTCAAGAAGCTGTTTGCTGGTATCAACTCTGTCTGTTTCGATGAGAAGTCTAAGCACATTACTGCAATGAA
GTCCTTGGAGGGAGAAGTTGTGCCATTCAAGAATAAGGTGCCCTTGTCCAATAACGTCGAAACCTGGCTGAACGATCTGGCCCTGGAGA
TGAAGAAGACCCTGGAGCAGCTGCTGAAGGAGTGCGTGACAACCGGACGCAGCTCTCAGGGAGCTGTGGACCCTTCTCTGTTCCCATCA
CAGATCCTGTGCTTGGCCGAACAGATCAAGTTTACCGAAGATGTGGAGAACGCAATTAAAGATCACTCCCTGCACCAGATTGAGACACA
GCTGGTGAACAAATTGGAGCAGTATACTAACATCGACACATCTTCCGAGGACCCAGGTAACACAGAGTCCGGTATTCTGGAGCTGAAAC
TGAAAGCACTGATTCTCGACATTATCCATAACATCGACGTGGTCAAGCAGCTGAACCAAATCCAAGTGCACACCACCGAAGATTGGGCC
TGGAAGAAGCAGTTGAGGTTCTACATGAAGTCCGACCACACCTGTTGCGTTCAGATGGTTGACAGCGAGTTCCAGTACACCTATGAGTA
CCAAGGAAATGCCAGCAAGCTCGTTTACACTCCACTCACTGACAAGTGTTACCTCACCTTGACACAGGCTATGAAGATGGGCCTGGGAG
GCAACCCATACGGTCCAGCTGGCACTGGTAAGACAGAGAGCGTTAAGGCACTCGGAGGTCTGCTGGGCAGGCAGGTCCTCGTGTTCAAC
TGTGATGAAGGAATCGACGTTAAGTCCATGGGAAGAATCTTTGTTGGCCTCGTTAAGTGTGGAGCTTGGGGTTGCTTCGACGAGTTCAA
CAGGCTGGAGGAATCTGTGCTGAGCGCCGTCTCTATGCAGATCCAGACCATCCAGGACGCATTGAAGAACCACAGGACCGTCTGCGAGC
TGTTGGGTAAGGAAGTGGAGGTGAACTCCAACTCCGGAATCTTCATCACAATGAATCCCGCAGGTAAAGGATATGGAGGAAGACAGAAA
CTCCCAGACAACCTGAAGCAGCTGTTCCGCCCAGTGGCTATGTCCCATCCAGACAATGAGCTGATCGCCGAAGTCATCCTCTATTCCGA
GGGATTCAAAGATGCTAAAGTTCTCTCCAGAAAGCTCGTGGCCATCTTCAATCTGTCAAGAGAACTCCTGACACCTCAGCAGCATTACG
ACTGGGGTCTGAGAGCCCTCAAGACCGTCCTGAGAGGTTCAGGAAATCTCCTCAGGCAGCTGAACAAGAGCGGTACAACACAGAATGCA
AATGAGAGCCACATTGTCGTCCAGGCTCTGAGGCTGAATACCATGTCAAAGTTCACATTCACAGACTGCACAAGATTTGACGCTCTGAT
TAAAGATGTGTTCCCTGGTATTGAACTCAAAGAAGTGGAGTATGACGAGCTGAGCGCCGCTTTGAAGCAGGTGTTTGAGGAGGCTAACT
ATGAGATTATCCCTAATCAGATCAAGAAAGCATTGGAACTGTATGAACAGCTGTGTCAGAGGATGGGAGTGGTGATTGTGGGCCCATCA
GGCGCAGGTAAGAGCACTCTCTGGAGAATGCTGAGAGCAGCACTGTGCAAGACTGGAAAGGTGGTGAAGCAATACACCATGAATCCC
```

Supplementary references

(1) Zadeh, J. N.; Steenberg, C. D.; Bois, J. S.; Wolfe, B. R.; Pierce, M. B.; Khan, A. R.; Dirks, R. M.; Pierce, N. A. NUPACK: Analysis and Design of Nucleic Acid Systems. *J Comput Chem* **2011**, *32* (1), 170–173. https://doi.org/10.1002/jcc.21596.

(2) Yang, Y.; Wang, J.; Shigematsu, H.; Xu, W.; Shih, W. M.; Rothman, J. E.; Lin, C. Self-Assembly of Size-Controlled Liposomes on DNA Nanotemplates. *Nat Chem* **2016**, *8* (5), 476–483. https://doi.org/10.1038/nchem.2472.

(3) Nafisi, P. M.; Aksel, T.; Douglas, S. M. Construction of a Novel Phagemid to Produce Custom DNA Origami Scaffolds. *Synth Biol* **2018**, *3* (1). https://doi.org/10.1093/synbio/ysy015.

(4) Douglas, S. M.; Marblestone, A. H.; Teerapittayanon, S.; Vazquez, A.; Church, G. M.; Shih, W. M. Rapid Prototyping of 3D DNA-Origami Shapes with CaDNAno. *Nucleic Acids Res* **2009**, *37* (15), 5001–5006. https://doi.org/10.1093/nar/gkp436.
